# Role of CD8^+^ regulatory T cells in organ transplantation

**DOI:** 10.4103/2321-3868.126086

**Published:** 2014-01-26

**Authors:** Jiyan Su, Qingfeng Xie, Yang Xu, Xian C. Li, Zhenhua Dai

**Affiliations:** 1 Immunobiology and Transplantation Research Center, Houston, Methodist Research Institute, Houston, Texas USA; 2 Center for Regenerative and Translational Medicine, Guangdong Provincial Academy of Chinese Medical Sciences, Guangdong Provincial Hospital of Chinese Medicine and Guangzhou University of Chinese Medicine School of Chinese Material Medica, Guangzhou, Guangdong, China; 3 Center for Regenerative and Translational Medicine, Guangdong Provincial Academy of Chinese Medical Sciences and the Second Affiliated Hospital of Guangzhou University of Chinese Medicine, 55 Nei Huan Xi Lu, College Town, Guangzhou, Guangdong, 510006 P.R. China

**Keywords:** Tolerance, transplantation, CD8^+^ regulatory T cell, immune regulation

## Abstract

CD8^+^ T cells are regulatory T cells (Tregs) that suppress both alloimmunity and autoimmunity in many animal models. This class of regulatory cells includes the CD8^+^CD28^−^, CD8^+^CD103^+^, CD8^+^FoxP3^+^ and CD8^+^CD122^+^ subsets. The mechanisms of action of these regulatory cells are not fully understood; however, the secretion of immunosuppressive cytokines, such as interleukin (IL)-4, IL-10 and transforming growth factor beta (TGF-β) as well as the direct killing of target cells via Fas L/Fas and the perforin/granzyme B pathways have been demonstrated in various models. Further studies are necessary to fully understand the mechanisms underlying the suppressive effects of Tregs and to provide experimental support for potential clinical trials. We recently observed that CD8^+^CD122^+^ Tregs more potently suppressed allograft rejection compared to their CD4^+^CD25^+^ counterparts, supporting the hypothesis that CD8^+^ Tregs may represent a new and promising Treg family that can be targeted to prevent allograft rejection in the clinic. In this review, we summarize the progress in the field during the past 7–10 years and discuss CD8^+^ Treg phenotypes, mechanisms of action, and their potential clinical applications; particularly in composite tissue transplants in burn and trauma patients.

## Introduction

Organ transplantation is the preferred treatment of choice for patients with end-stage organ failure. However, the mortality and mobility associated with broad immunosuppression in transplant patients remains a significant barrier to the actual therapeutic potential of organ transplantation. The recent emergence of composite tissue transplantation, which is the treatment of choice for patients suffering from severe burns and trauma, presents new challenges and opportunities for long-term transplant survival without drug-associated toxicities. Induction of immune tolerance is essential for avoiding pathogenic immune responses to self-antigens and alloantigens. Tolerance is also an ideal method of weaning patients off immunosuppressive drugs. It has been well-established that regulatory T cells (Tregs) are critical for maintaining immune tolerance by suppressing both autoimmunity and alloimmunity. There are multiple cell types in the immune system that exhibit immunosuppressive properties. In addition to the much publicized CD4^+^CD25^+^Foxp3^+^ Tregs, CD8^+^ Tregs have emerged as another key player in immune regulation. Early studies showed that CD8^+^ T cells could suppress immune responses, thereby functioning as suppressor cells,[12] and tremendous progress has occurred regarding their suppression of alloimmunity and mechanisms of action. For example, Liu *et al.*, demonstrated that CD8^+^CD28^−^ T cells inhibited T helper alloreactivity in an major histocompatibility complex (MHC) class I-restricted manner.[3] These CD8^+^ Tregs also induced xenoreactive CD4^+^ T-cell anergy. [4] Human CD8^+^ Tregs inhibited graft-versus-host disease (GVHD) in humanized mice.[5] Additionally, either donor-specific or induced CD8^+^FoxP3^+^ Tregs facilitated skin allograft survival in certain models.[6] Moreover, antigen-induced CD8^+^CD103^+^ Tregs have been shown to suppress alloreactive effector T cell function.[7] More importantly, several recent studies have shown that CD8^+^CD122^+^ T cells are also potent Tregs that inhibit both alloimmunity and autoimmunity.[8,19] In particular, we have recently found that in an islet transplant model, naturally occurring CD8^+^CD122^+^ Tregs in the periphery more potently suppressed allograft rejection than their CD4^+^CD25^+^ counterparts. [20] Therefore, CD8^+^ Tregs may represent a new and promising Treg family that can be targeted to treat autoimmune diseases and allograft rejection in the clinic. Here, we review the most recent progress in the field and discuss potential clinical applications for the induction of tolerance to composite tissue transplants.

**Table 1:** 

Access this article online	
**Quick Response Code:**	**Website:** www.burnstrauma.com
	**DOI:** 10.4103/2321-3868.126086

## CD8^+^CD28^−^ Tregs

Previous studies have demonstrated that CD8^+^CD28^−^ T cells are immunosuppressive.[18] Liu *et al.*, originally reported the specific suppression of T helper alloreactivity by MHC class-I-restricted CD8^+^CD28^−^ T cells,[3] which were generated by multiple rounds of *in vitro* allostimulation of peripheral blood mononucleocytes. These MHC class-I-restricted CD8^+^CD28^−^ T cells induced xenoreactive human CD4^+^ T-cell anergy upon specific recognition of MHC class-I antigens.[4] Adoptive transfer of CD8^+^CD28^−^ cells resulted in profound inhibition of corneal xenograft rejection.[21] Qa-1-restricted CD8^+^CD28^−^ T cells regulated the reactivation of both T cells and natural killer T (NKT) cells.[22] Human xenospecific CD8^+^CD28^−^ Tregs inhibited T helper cell responses to porcine aortic endothelial cells by suppressing necrosis factor (NF)-kappa B activity in porcine antigen-presenting cells (APCs).[23] Allospecific and induced CD8^+^CD28^−^ FoxP3^+^ Tregs induced immunoglobulin-like transcript (ILT)3^+^/ILT4^+^ tolerogenic endothelial cells or APCs, down-regulated the expression of costimulatory (CD80/CD86) and adhesion (CD54/CD58) molecules, thereby suppressing alloimmune responses.[23–26] Although induced CD8^+^CD28^−^ Tregs expressed FoxP3,[24] natural CD8^+^CD28^−^ Tregs were FoxP3-negative.[27] The induced, but not natural, CD8^+^CD28^−^ Tregs expressed glucocorticoid-induced tumor necrosis factor receptor-related protein (GITR) and cytotoxic T-cell antigen 4 (CTLA4), whereas both Tregs expressed CD62L[27,28] [Figure 2]. Moreover, graft function was maintained with minimal or no immunosuppression in patients that had circulatory CD8^+^CD28^−^ Tregs.[29] The expansion of CD8^+^CD28^−^ Tregs was associated with the reduced occurrence of acute or chronic rejection.[30] The adoptive transfer of CD8^+^CD28^−^ Tregs from tolerized liver transplant recipients alleviated acute allograft rejection in rats.[31] Donor-specific T cell vaccination induced CD4^+^CD25^+^ or CD8^+^CD28^−^ Tregs that promoted transplant tolerance in new recipient mice that received a cardiac allograft.[32] Taken together, mounting evidence suggests that CD8^+^CD28^−^ cells are Tregs that can either suppress allograft rejection or promote allograft tolerance. CD8^+^CD28^−^ Tregs are the primary effectors of transplant tolerance and act on the endothelium and antigen presenting cells (APCs) to induce a tolerogenic phenotype and inhibit CD4^+^ T helper cell alloreactivity.

## CD8^+^CD103^+^ Tregs

**Figure 1:**
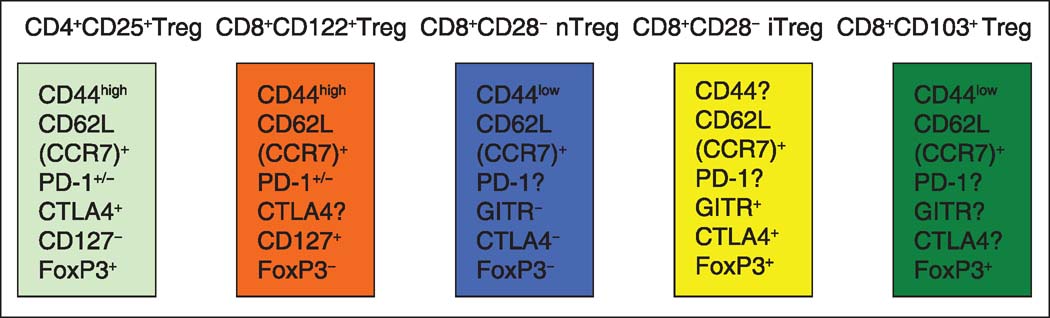
CD8^+^ regulatory T cell (Treg) phenotypes. Natural CD8^+^CD122^+^ Tregs are CD44^high^CD62L^+^CD127^+^ and partially PD-1-positive (PD-1^+/−^) but are FoxP3-negative. Natural CD8^+^CD28^−^ Tregs (nTreg) are CD44^low^CD62L^+^ but GITR-CTLA4-FoxP3^−^; whereas, induced CD8^+^CD28^−^ Tregs (iTreg) are CD44^high^CD62L^+^, but GITR^+^CTLA4^+^FoxP3^+^. CD8^+^CD103^+^ Tregs are CD44^low^CD62L^high^FoxP3^+^. GITR = Glucocorticoid-induced tumor necrosis factor receptor-related protein, CTLA4 = cytotoxic T-cell antigen 4.

It has been shown that a CD8^+^ T cell population lacking CD44 but expressing CD103 produced transforming growth factor beta (TGF-β), inhibit CD4^+^ proliferation *in vitro*, and attenuate adoptively transferred ileitis *in vivo*, most likely by counteracting the proinflammatory role of the CD44^high^ subset of T cells.[33] Therefore, these CD8^+^ Tregs exhibit a naive T cell phenotype (CD8^+^CD103^+^CD44^low^CD62L^high^), as shown in Figure 1. In contrast, allostimulated CD8^+^CD103^+^ Tregs displayed limited effector functions, such as cytotoxicity and interferon (IFN)-γ production. Instead, CD8^+^CD103^+^ Tregs suppressed alloreactive effector T cells.[7] Lu *et al.*, demonstrated that CD8^+^CD103^-^ T cells differentiated into CD8^+^CD103^+^FoxP3^+^ T cells after *in vitro* stimulation with either alloantigens or transforming growth factor (TGF)-β and that the number of CD8^+^CD103^+^ Tregs increased in spontaneously tolerant recipients of a liver allograft.[34] Rapamycin not only promoted the *in vitro* generation of CD8^+^CD103^+^ Tregs after allostimulation, but also enhanced their suppressive capacity.[35] However, a study by Feng *et al.*, found that CD103 expression by CD8^+^ T cells was required for the destruction of islet allografts because CD8^+^CD103 T cells failed to infiltrate the islet grafts,[36] indicating that CD103 may be an effector but not a regulatory molecule. Therefore, further studies are necessary to better define the role of CD103 in alloreactive CD8^+^ T cell functions.

## CD8^+^CD122^+^ Tregs

Recent studies have shown that naturally occurring CD8^+^CD122^+^ T cells control T cell homeostasis and play an important role in the suppression of autoimmune diseases.[8] The transfer of CD8^+^CD122^+^ Tregs significantly improved the clinical symptoms of experimental autoimmune encephalomyelitis (EAE), indicating a role for CD8^+^CD122^+^ Tregs in the recovery phase of EAE.[37] Brain DCs expressing the B7-H1 molecule recruit CD8^+^CD122^+^ Tregs to the inflammatory site, which causes a decrease in the clinical EAE peak values.[38] Notably, CD8^+^CD38^+^CD122^+^ Tregs inhibited effector CD4^+^ T cell proliferation in an antigen-nonspecific manner and reduced the clinical score of murine EAE as well as delayed disease occurrence.[39] A recent study showed that CD8^+^CD122^+^ T cells also played a regulatory role in EAE models of HLA-DR3 transgenic mice; whereas, CD8^+^CD122 T cells had an opposite role.[16] Furthermore, CD8^+^CD122^+^ Tregs suppressed other autoimmune diseases in many animal models. The depletion of CD8^+^CD122^+^ T cells increased the incidence of autoimmune Graves’hyperthyroidism in a mouse model,[40] suggesting that CD8^+^CD122^+^ T cells play an essential role in the inhibition of autoimmune hyperthyroidism. B6-Yaa mutant mice developed systemic lupus erythematous-like disease in association with a defect in CD8^+^CD122^+^ Treg activity, suggesting that the Treg subset may be utilized to treat systemic lupus erythematous-like autoimmune disease.[15] Therefore, in multiple experimental animal models, CD8^+^CD122^+^ Tregs play an important role in the suppression of various autoimmune diseases.

Recent studies have also demonstrated that CD8^+^CD122^+^ Tregs play a role in suppressing alloimmune responses. We originally reported that CD8^+^CD122^+^ Tregs suppressed murine allograft rejection.[17] We also found that the PD-1^+^ component of these Tregs were more effective than the unfractionated CD8^+^CD122^+^ Treg population;[17] whereas, antigen-specific CD8^+^CD122^+^PD-1 cells were memory T cells that could respond to a previously encountered antigen quickly and efficiently. Furthermore, we demonstrated that CD8^+^CD122^+^ Tregs were more potent in suppressing allograft rejection than their CD4^+^CD25^+^ counterparts,[20] suggesting that CD8^+^CD122^+^ Tregs may be a better target for the treatment of allograft rejection. In summary, CD8^+^CD122^+^ Tregs regulate both autoimmunity and alloimmunity and may participate in immune regulation *in vivo* for various diseases.

CD8^+^CD122^+^ Tregs and the classical CD4^+^CD25^+^ Tregs both express the interleukin (IL)-2 receptor. Specifically, CD122 is the β subunit of the IL-2 receptor on T cells; whereas, CD25 is the α subunit of the IL-2 receptor.[41] To identify more effective Tregs for potential clinical applications, we evaluated the efficacy of naturally arising CD8^+^CD122^+^ vs CD4^+^CD25^+^ Tregs for suppressive activities. Surprisingly, we found that CD8^+^CD122^+^ Tregs were much more efficient in the suppression of allograft rejection and underwent faster homeostatic proliferation than their CD4^+^CD25^+^ counterparts.[20] In addition, CD8^+^CD122^+^ Tregs produced significantly more IL-10 and were more effective in the suppression of *in vitro* T cell proliferation than their CD4^+^CD25^+^ counterparts. Importantly, adoptive transfer of CD8^+^CD122^+^ Tregs but not CD4^+^CD25^+^ Tregs, together with the infusion of recombinant IL-15, significantly delayed allograft rejection in normal wild-type mice. In contrast, the transfer of CD4^+^CD25^+^ Tregs with the administration of either recombinant IL-2 or IL-15 did not significantly prolong islet allograft survival.[20] We hypothesized that IL-2 administration promoted the expansion of both CD4^+^CD25^+^ Tregs and conventional effector T cells, which did not alter the overall immune balance; whereas, the administration of IL-15 enhanced the expansion and function of CD8^+^CD122^+^ but not CD4^+^CD25^+^ Tregs. Therefore, naturally occurring CD8^+^CD122^+^ Tregs appear to be a more promising target for suppressing allograft rejection than their CD4^+^CD25^+^ counterparts. Further studies are warranted to improve CD8^+^CD122^+^ Treg therapies and provide additional experimental data for clinical trials.

## Mechanisms of action

**Figure 2:**
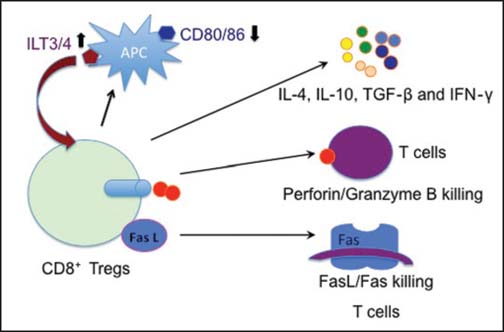
Mechanisms underlying CD8^+^ Treg suppression. Mechanisms responsible for CD8^+^ Treg suppression include immunosuppressive cytokines and the killing of target T cells via the perforin/granzyme B and Fas L/Fas pathways as well as the downregulation of CD80/CD86 but the upregulation of immunoglobulin-like transcript 3/4 (ILT3/4) that, in turn, promotes CD8^+^ Treg expansion antigen presenting cell (APC).

The mechanisms underlying CD8^+^ Treg suppression are not fully understood. However, multiple mechanisms are likely to be involved, including the killing or direct lysis of target cells, the induction of CD4^+^ T cell anergy, and the secretion of immunosuppressive cytokines and molecules. Different subsets of CD8^+^ Tregs may utilize distinct mechanisms. Studies by Cantor’s group have demonstrated that perforin-mediated cytotoxicity is required for the suppressive activity of Qa-1-restricted CD8^+^ Tregs;[42] whereas, others have shown that CD11c^+^CD8^+^ Tregs can directly kill activated CD4^+^ T cells through the Fas ligand-Fas death pathway[43] [Figure 2]. CD8^+^CD28^−^ Tregs induced xenoreactive human CD4^+^ T-cell anergy[4] and also downregulated expression of the costimulatory molecules CD80/CD86 on APCs,[23] which indirectly block T cell priming by APCs. TGF-β expanded CD8^+^CD103^+^ Tregs displayed cytotoxicity towards allospecific effector T cells through cell-to-cell contact.[7] Zheng *et al.*, demonstrated that alloantigen-specific suppression by human CD8^+^ Tregs was partially dependent on IL-10, TGF-β, GITR and CTLA-4 expression.[44] CD8^+^CD28^−^ Tregs induced ILT3^+^/ILT4^+^ expression on endothelial cells or APCs and downregulated costimulatory (CD80/CD86) and adhesion (CD54/CD58) molecules, resulting in reduced alloreactivity.[23,26] The increased ILT3^+^/ILT4^+^ expression, in turn, promoted the differentiation of CD8^+^CD28^−^ FoxP3^+^ Tregs,[24] reinforcing their suppressive capacity. Moreover, ILT3 directly induced CD4^+^ Th cell anergy and suppressed the differentiation of IFN-γ-producing effector CD8^+^ CTL.[45]

The mechanisms of action for CD8^+^CD122^+^ Treg suppression are also not well-defined. As shown in Figure 2, IL-10 produced by CD8^+^CD122^+^ Tregs appears to be a primary mechanism responsible for suppression.[9,17,46] Endharti *et al.*, presented the first data showing that CD8^+^CD122^+^ Tregs suppressed IFN-γ production and the proliferation of CD8^+^ T cells by producing IL-10 *in vitro*.[9] CD8^+^ Tregs also recognized activated T cells via the interaction of the MHC class I-αβ TCR and regulated target T cells by producing IL-10.[46] We also observed that the suppression of allograft rejection by IL-10-deficient CD8^+^CD122^+^ Tregs was largely diminished.[17] Activated CD8^+^CD122^+^ Tregs from RasGRP1(-/-) mice synthesized IL-10 and inhibited the proliferation of CD8^+^CD122^−^ T cells.[10] However, IL-10 did not account for all mechanisms underlying CD8^+^CD122^+^ Treg suppression.[17] Other mechanisms, in addition to IL-10 production, may be involved in CD8^+^CD122^+^ Treg suppression. In particular, CD8^+^CD122^+^ Tregs also released IFN-γ and TGF-β that suppressed CD4^+^ T cell activation.[16] It remains to be determined whether the perforin/granzyme B pathway is also involved in regulating effector T cells by CD8^+^CD122^+^ Tregs. Despite these findings, further studies are required to fully identify the mechanisms responsible for the suppression of alloimmunity or autoimmunity.

## Conclusion

Many recent studies have shown that the CD8^+^ component of T cells can also serve as Tregs *in vitro* and *in vivo*, although relatively little is known about these cells compared to the conventional CD4^+^CD25^+^ Tregs. CD8^+^ cells suppress both alloimmune responses and autoimmunity in many animal models. Multiple subsets of CD8^+^ Tregs have been identified thus far, including the CD8^+^CD28^−^, CD8^+^CD103^+^, CD8^+^FoxP3^+^ and CD8^+^CD122^+^ populations. The mechanisms of action of these cells are not well understood because of their plasticity and heterogeneity. Further investigation is necessary to fully understand the distinct mechanisms underlying their suppression as well as the mechanism by which CD8^+^ Tregs interact with other types of Tregs in the induction and maintenance of immune tolerance. These studies will help lay the groundwork for potential clinical trials. Most importantly, our new studies reveal that CD8^+^CD122^+^ Tregs more potently suppress allograft rejection than their CD4^+^CD25^+^ counterparts. Therefore, CD8^+^ Tregs likely represent a new and promising Treg family that can be targeted to suppress allograft rejection or induce long-term allograft survival or tolerance in the clinic. This will undoubtedly benefit transplant patients, including those with composite tissue transplants because of trauma and burns.
